# Free-Energy Profiles of Confined Reactions: Influence of Confinement Type and Challenges for Metadynamics Methods

**DOI:** 10.1021/acsphyschemau.5c00151

**Published:** 2026-04-24

**Authors:** Michelle Ernst, Jürg Hutter

**Affiliations:** † Institute of Geological Sciences, 27210 University of Bern, Baltzerstrasse 1 + 3, 3012 Bern, Switzerland; ‡ Department of Chemistry, 27217 University of Zurich, 8057 Zurich, Switzerland

**Keywords:** molecular dynamics, metadynamics, free energy surface, confinement, Diels−Alder, S_N_2, carbon nanotubes

## Abstract

Metadynamics enables the reconstruction of free-energy surfaces from molecular dynamics; however, its application is often challenging because numerous methodological choices can influence the results. Here, we focus on how geometric confinement reshapes reactant and product basins and thereby contributes to catalytic effects. Our analysis is based on two prototypical reactions: a symmetric S_N_2 fluorine-substitution reaction, CH_3_F + F^–^ *⇌* F^–^ + CH_3_F and a Diels–Alder cycloaddition. We compare several metadynamics approaches: well-tempered metadynamics, on-the-fly probability-enhanced sampling (OPES), OPES-Explore, and a hybrid OPES + OPES-Explore scheme, and we examine two types of confinements: First, we analyze the influence of methodological confinement introduced by restraining walls, a commonly used but seldom assessed component of enhanced sampling simulations. Second, we examine chemical confinement inside carbon nanotubes of varying diameters. We find that the restraining wall does not alter the activation free energy of the studied reactions but significantly affects the widths of the reactant and product basins. In contrast, confinement inside carbon nanotubes changes the barrier height by enforcing axial alignment of the reactants in the S_N_2 reaction and by restricting the transition-state and product geometries in the Diels–Alder case. Concerning the metadynamics methods, we found different convergence behavior and sampling quality, even for these simple reactions. The results highlight how both physical and methodological confinement can influence the outcome of enhanced-sampling simulations, and they underscore the need for careful choice of metadynamics methods for reactions in restricted environments.

## Graphical abstract

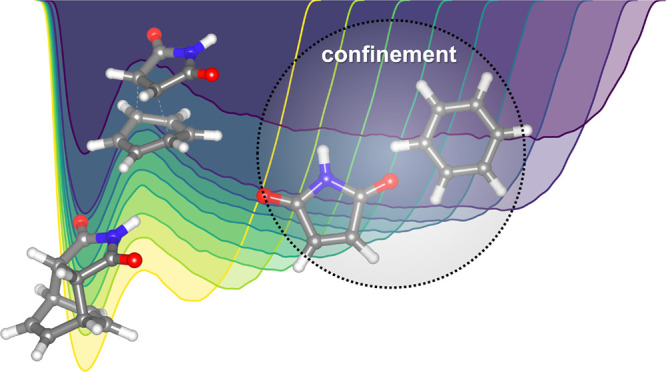

## 1 Introduction

Catalysis in confined spaces is a fundamental principle of chemical reactivity. Nature provides numerous examples of catalytic processes occurring in constrained environments. Enzymes achieve exceptionally high catalytic activity for specific reactions, surpassing the performance of synthetic catalysts.^1^ This remarkable efficiency arises from the precisely structured microenvironments within the enzyme active sites, where steric constraints, electrostatic stabilization, and specific solvation effects shape the free energy landscape of the reaction. These principles are not exclusive to biological systems, but can be mimicked in synthetic porous materials such as zeolites, metal–organic frameworks (MOFs), covalent organic frameworks (COFs), and carbon nanotubes (CNTs).^2^ The well-defined porous structure of these materials enables selective binding and activation of reactants, leading to enhanced catalytic activity and reaction selectivity. Zeolites, for instance, have been extensively used in industrial catalysis due to their shape-selective properties,^3,4^ while MOFs and COFs offer tunable pore sizes and functionalities, making them attractive platforms for designing advanced catalytic systems.^5^

Confinement in these systems exerts a wide range of effects that change molecular interactions and reaction mechanisms, and often significantly improve catalytic efficiency. A key effect is the selective adsorption of reactants from complex mixtures, either because only certain molecules are small enough to enter confined spaces,^6^ or because specific reactants exhibit stronger interactions with pore walls.^7^ This selective adsorption can lead to an increased local reactant concentration, effectively increasing the probability of reaction events.^8^ Confinement also alters guest diffusion, which in turn influences reactant mobility and transport within the pores.^9^ Additionally, spatial constraints within confined environments impose restrictions on molecular motion and conformational flexibility, thereby influencing the entropic contributions to reaction kinetics.^10^ The stabilization or destabilization of transition states within confined spaces represents another critical factor in confined catalysis. Some transition states may be too large to form within the restricted environment, while others may be stabilized or destabilized, changing the reaction pathway and activation energy.^11^ Moreover, confinement can impact product formation and their separation from the reaction mixture.^12^ In addition, it alters the dynamics^13^ and electronic structure^14^ of the solvent. Other confinement-induced effects include local electric field modifications,^15^ chiral discrimination,^16^ and cooperative interactions between multiple active sites within the confined space.^17^ Collectively, these factors reshape the electronic, thermodynamic, and kinetic landscape of chemical reactions, leading to changes in reaction selectivity, overall rates, and catalytic efficiency. A thorough understanding of confinement effects is therefore essential for accurately describing reaction mechanisms, interpreting experimental observations, and guiding the rational design of more efficient catalysts.

Yet, despite significant experimental and computational progress, our understanding of how confinement shapes free energy landscapes and reaction dynamics remains incomplete, owing to the complexity and interdependence of the underlying effects.

Here, we present a molecular dynamics study combined with metadynamics to investigate the impact of confinement on free energy landscapes and reaction behavior. This approach allows us to isolate and quantify specific confinement-induced effects while consistently accounting for electronic structure and nuclear dynamics over time. Metadynamics is an enhanced sampling method that accelerates rare events by applying a bias potential along selected collective variables (CVs), thereby enabling the reconstruction of the underlying free energy surfaces (FES). Two prototypical reactions were considered: a bimolecular nucleophilic substitution (S_N_2) and a Diels–Alder reaction. As a first step, we examined the effect of constraining walls, which are frequently employed in metadynamics simulations but rarely subjected to critical evaluation. In this context, we also tested several variants of metadynamics, including recent developments. Subsequently, we confined the reactions within carbon nanotubes of varying diameters to systematically probe how nanoconfinement modifies their free energy landscapes and reaction dynamics (cf. Figure 1).

**Figure 1:**
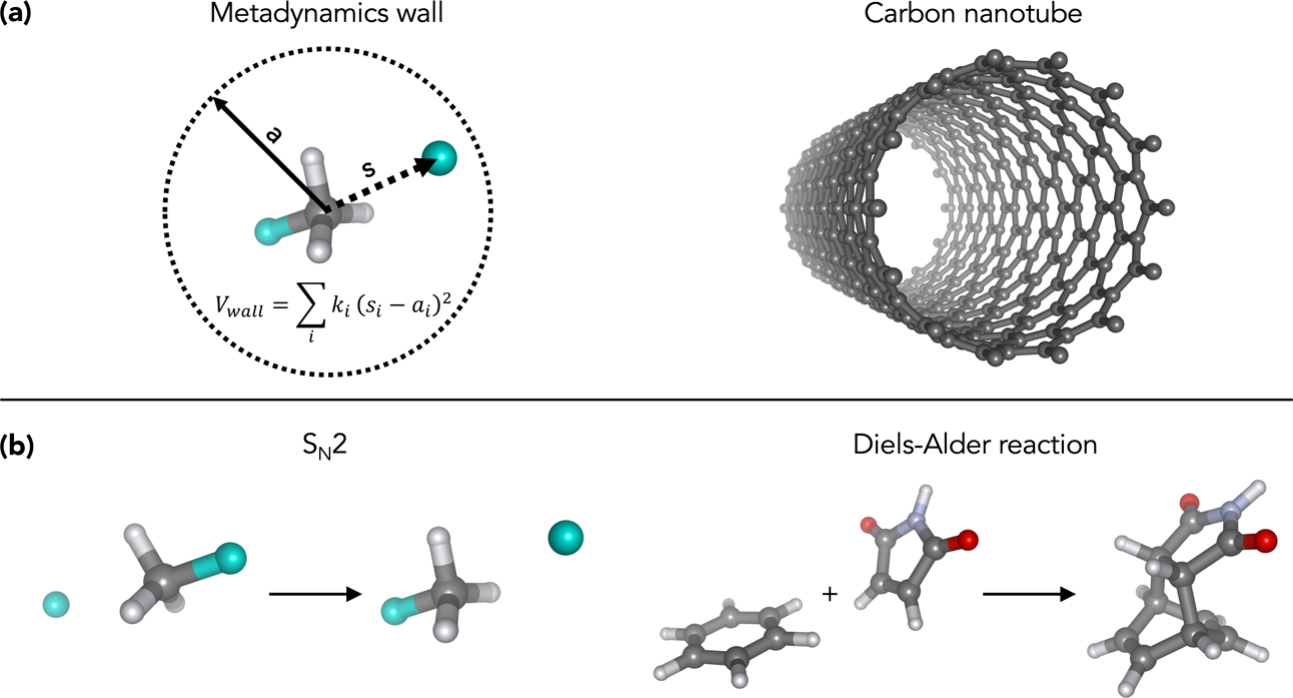
Overview of the systems and reactions examined in this work. (a) Confinement types: artificial restraining walls frequently applied in metadynamics simulations and carbon nanotubes of tunable diameters. (b) Investigated reactions: bimolecular nucleophilic substitution (S_N_2) and Diels–Alder cycloaddition.

## 2 Theoretical Background

### 2.1 Metadynamics

In the time scales accessible to electronic-structure-based molecular dynamics, energy barriers such as those associated with many chemical reactions are rarely overcome. Therefore, enhanced sampling techniques are required to accelerate rare events.^18^ In this work, we employ metadynamics, which introduces a repulsive bias potential acting on a set of collective variables chosen such that they distinguish the relevant metastable states.^19^ The bias potential is constructed incrementally by depositing Gaussian hills throughout the simulation. As bias accumulates, the system is increasingly discouraged from revisiting previously sampled configurations along the chosen CVs, thereby enabling the exploration of configurational regions that would otherwise remain inaccessible.

In standard metadynamics, the history-dependent bias potential at time t on the collective variable **s** is given by a sum over all previously deposited Gaussian hills:

$$V_{t}(s)=\overset{n}{\underset{k=1}{\sum }}W\;\exp\left(-\overset{N_{\mathrm{CV}}}{\underset{i=1}{\sum }}\frac{(s_{i}-s_{i,k}{)}^{2}}{2\sigma_{i}^{2}}\right)$$ 1

Here, *W* is the Gaussian height, σ_*i*_ is the width along the *i*-th CV, and *N*_CV_ is the number of collective variables. *k* counts the number of added hills and does not correspond to molecular dynamics steps. *s*_*i*_ denotes the current value of the i-th collective variable, while *s*_*i*,*k*_ is its value at the time the *k*-th hill was deposited.

If the simulation is run for a sufficiently long time, the cumulative bias cancels out the local minima of the free energy surface. At this stage, the negative of the bias provides an accurate estimate of the free energy surface, up to an additive constant:

F(s)=−V(s)+C 2

This principle is illustrated schematically in Figure 1e–h of ref 20.

Well-tempered metadynamics (WT-MetaD) refines this approach by gradually decreasing the height of the added Gaussian hills according to the bias already accumulated during the simulation. This adaptive bias deposition prevents oversampling and accelerates convergence toward the true free energy surface. The time dependent bias potential is given by

$$\begin{array}{cc} V_{t}(s) & =\overset{n}{\underset{k=1}{\sum }}\exp\left(-\frac{1}{\gamma -1}\beta V_{k-1}(s_{k})\right)H_{o}\;\exp\left(-\overset{N_{\mathrm{CV}}}{\underset{i=1}{\sum }}\frac{(s_{i}-s_{i,k}{)}^{2}}{2\sigma_{i}^{2}}\right)\\ & =\overset{n}{\underset{k=1}{\sum }}H_{k}\;\exp\left(-\overset{N_{\mathrm{CV}}}{\underset{i=1}{\sum }}\frac{(s_{i}-s_{i,k}{)}^{2}}{2\sigma_{i}^{2}}\right) \end{array}$$ 3

where *H*_0_ is the height of the initial Gaussian, γ is the chosen bias factor, and β is proportional to the inverse temperature (β = 1/(*k*_b_ *T*)). The term *H*_*k*_ represents the effective height of the *k*-th Gaussian after bias scaling.

At convergence, the free energy surface is related to the bias by

$$F\left(s\right)=-\frac{\gamma }{\gamma -1}V\left(s\right)+C$$ 4

and the resulting sampled ensemble corresponds to a smoothed version of the unbiased distribution, in which the free energy barriers are effectively reduced by a factor of γ. For a detailed review, see ref 18.

#### 2.1.1 On-the-Fly Probability-Enhanced Sampling

A recent advancement among the metadynamics methods is the on-the-fly probability enhanced sampling (OPES), which differs from WT-MetaD in how the bias potential is constructed. Instead of directly accumulating repulsive Gaussians in the collective variable space, OPES builds the bias iteratively based on an on-the-fly estimate of the equilibrium probability distribution along the chosen CVs.^21^

OPES aims to modify the sampled probability distribution *p*(**s**) so that it approaches a chosen target distribution *p* ^tg^(**s**). In the well-tempered formulation of OPES, this target distribution is defined as

$$p^{\mathrm{tg}}\left(s\right)\propto {\left[p_{0}\left(s\right)\right]}^{1/\gamma }$$ 5

where *p*_0_(**s**) denotes the original (unbiased) distribution and γ is the bias factor. This target represents a flattened version of the original distribution that preserves its overall shape but lowers the height of the free energy barriers by a factor of γ, thereby facilitating transitions between metastable states. Compared to well-tempered metadynamics, OPES achieves faster and more stable convergence because the bias is constructed from an on-the-fly estimate of the probability distribution rather than from cumulative hill deposition.

To achieve this, the required bias potential is given by

$$V(s)=-\frac{1}{\beta }\log\;\frac{p^{\mathrm{tg}}(s)}{p_{0}(s)}=-\frac{1}{\beta }\log\;\frac{p_{0}(s{)}^{1/\gamma }}{p_{0}(s)}=\left(1-\frac{1}{\gamma }\right)\frac{1}{\beta }\log\;p_{0}(s)$$ 6

Since the exact *p*_0_(**s**) is unknown, it is estimated on-the-fly as *p̂*_*n*_(**s**) (see eq 11) and normalized over the region of CV space explored up to step n, denoted Ω_*n*_, using

$$Z_{n}=\frac{1}{\left|\Omega_{n}\right|}\int_{\Omega_{n}}{\hat{p}}_{n}(s)ds$$ 7

Inserting this into the bias expression and adding a regularization term ϵ to ensure that the argument of the logarithm is strictly positive yields

$$V_{n}(s)=\left(1-\frac{1}{\gamma }\right)\frac{1}{\beta }\log\left(\frac{{\hat{p}}_{n}(s)}{Z_{n}}+\epsilon \right)$$ 8

The parameters ϵ and γ are controlled by the user-specified barrier parameter Δ*E* as

γ=βΔE 9

$$\epsilon =\exp\left[-\frac{\beta \Delta E}{1-\frac{1}{\gamma }}\right]$$ 10

In practice, the estimate ${\hat{p}}_{n}(s)$ is obtained using a reweighted kernel density estimation scheme. As the simulation progresses, Gaussians are periodically deposited in CV space, and the unbiased distribution is estimated as

$${\hat{p}}_{n}(s)=\frac{\sum_{k=1}^{n}\omega_{k}G(s,\;s_{k})}{\sum_{k=1}^{n}\omega_{k}}$$ 11

where *G*(**s**, **s**_*k*_) is a Gaussian kernel centered at the previously sampled point **s**_*k*_, and the weights are computed from the accumulated bias as

$$\omega_{k}\propto \exp\left[\beta V_{k-1}\left(s_{k}\right)\right]$$ 12

This formulation allows the bias to adapt naturally as the underlying distribution becomes better resolved over time.

OPES-Explore is a variant of the OPES method that prioritizes exploration over rapid convergence.^22^ Like OPES, it aims to sample from a well-tempered target distribution.

However, instead of estimating the unbiased distribution *p*_0_(**s**) through reweighting, OPES-Explore constructs the bias directly from an unweighted estimate of the well-tempered distribution:

$${\hat{p}}_{n}^{\mathrm{WT}}(s)=\frac{1}{n}\sum_{k=1}^{n}G_{k}(s,\;s_{k})$$ 13

where *G*(**s**, **s**_*k*_) is a Gaussian kernel centered at the sampled CV value **s**_*k*_. The corresponding bias potential is then

$$V_{n}(s)=(\gamma -1)\frac{1}{\beta }\log\left(\frac{{\hat{p}}_{n}^{\mathrm{WT}}(s)}{Z_{n}}+\epsilon \right)$$ 14

Because this estimate does not rely on reweighting, the resulting bias is more responsive to recent sampling, which promotes faster transitions between metastable states and improves the exploration efficiency. This is particularly advantageous when the chosen CVs do not fully resolve the slow degrees of freedom.

To address the limitations of OPES and OPES-Explore when used independently, a combined approach has been proposed that combines the strengths of both methods.^23^ In this scheme, two bias potentials are applied simultaneously: a slowly evolving OPES bias *V*_*n*_^o^(**s**) designed for accurate convergence through reweighting, and a rapidly fluctuating OPES-Explore bias *V*_*n*_^e^(**s**), which promotes enhanced exploration. The total bias applied to the system is the sum of the two. The OPES component is updated using a reweighted probability distribution that implicitly accounts for the effect of the OPES-Explore bias.

More generally, recent methodological developments aim to improve the exploration of complex free-energy surfaces by coupling enhanced sampling with machine-learned collective variables.^24,25^

#### 2.1.2 Restraining Walls

To prevent CVs from exceeding predefined values, restraining walls can be applied. For an upper wall, the bias acts when the CV value is greater than *a*_*i*_. The form of the wall bias is

$$V_{\mathrm{wall}}=\underset{i}{\sum }k_{i}{\left(s_{i}-a_{i}\right)}^{e_{i}}$$ 15

where *s*_*i*_ is the current value of the CV, *k*_*i*_ is the force constant, *a*_*i*_ is the wall position, and *e*_*i*_ defines the functional form of the potential. In this work, the parameters were fixed to *e*_*i*_ = 2, resulting in a simple quadratic restraining potential (harmonic wall).

## 3 Simulation Details

### 3.1 Molecular Dynamics

All electronic structure based molecular dynamics simulations were performed using CP2K^26^ version 2024.3. We employed the GFN1 extended tight-binding (GFN1-xTB) method^27^ as implemented in CP2K. All simulations were carried out in the NVT ensemble employing the canonical sampling through velocity rescaling (CSVR) thermostat^28^ with a relaxation time constant of 200 fs, maintaining the temperature at approximately 300 K within a tolerance of 50 K. Since no solvent is present, equilibration runs were not required. A time step of 0.5 fs was used throughout, and a cubic simulation box of 20 Å per side was applied for systems without carbon nanotubes.

### 3.2 Static Reaction Profile

Static reaction profiles, including reactant, transition state (TS), and product energies, were computed using Gaussian16 ^29^ interfaced with the external xTB plugin,^30^ employing a TS geometry obtained from a preceding PBE/cc-pVDZ calculation. Thermodynamic properties were obtained from vibrational frequency calculations performed with the xTB method.

### 3.3 Metadynamics

All metadynamics simulations were carried out using PLUMED version 2.9.0 ^31^ as a plugin in CP2K. We compared several metadynamics approaches: well-tempered metadynamics,^19^ on-the-fly probability enhanced sampling,^21^ OPES-Explore,^22^ and the recently proposed hybrid method that combines OPES with OPES-Explore.^23^

For the S_N_2 reaction, Gaussian hills were deposited every 120 MD steps, while for the Diels–Alder reaction one hill was added every 200 MD steps. In WT-MetaD, the Gaussian width was set to σ = 0.05 Å, the initial hill height to *W* = 5 kJ/mol, and the bias factor γ was chosen as 50 for *S*_N_2 and 100 for the Diels–Alder reaction. For OPES based simulations, the barrier parameter Δ*E* was set to 80 kJ/mol for S_N_2 and 200 kJ/mol for the Diels–Alder reaction. The force constant of the restraining wall (cf. eq 15) was fixed to 150 kJ mol^–1^ Å^–2^ in all cases. Free energy surfaces were reconstructed using the sum_hills utility for WT-MetaD and the fes_from_reweighting python script for OPES based simulations.^32^

### 3.4 Reaction Specificities and Choice of Collective Variables

#### 3.4.1 S_N_2 Reaction with Fluorine

The S_N_2 reaction CH_3_F + F^–^ → CH_3_F + F^–^ (cf. Figure 1b) produces a symmetric free energy surface, making it well-suited for benchmarking the performance and convergence of enhanced sampling methods and the effect of confinement.

The chosen CV is defined as 0.5·*d*_1_ – 0.5·*d*_2_, where *d*_1_ is the distance between the carbon atom and the leaving fluorine atom, and *d*_2_ is the distance between the carbon and the incoming fluoride nucleophile.

#### 3.4.2 Diels–Alder Reaction between Benzene and Maleimide

The Diels–Alder reaction was selected as a prototypical reaction for studying confinement effects on pericyclic processes and their free energy landscapes. This reaction is widely used as a benchmark in computational chemistry and experimentally has been reported to exhibit confinement-enhanced reactivity in porous materials.^33^ To retain the essential features of Diels–Alder reactivity at minimal computational cost, benzene and maleimide were chosen as simple model reactants. This provides a representative yet computationally tractable model for examining Diels–Alder reactivity under confinement. Together with the S_N_2 reaction, it forms a minimal yet chemically relevant set of test systems, involving few atoms and only common elements.

For this reaction, the collective variable is defined as 0.5·*d*_1_ + 0.5·*d*_2_, where *d*_1_ and *d*_2_ correspond to the distances between the two pairs of carbon atoms that form the new C–C bonds in the course of the reaction. Restraining walls were applied to each distance individually, using identical wall positions for *d*_1_ and *d*_2_.

### 3.5 Carbon Nanotubes

To introduce confinement not only through metadynamics walls (as described in Section 2.1.2) but also through chemical confinement, carbon nanotubes were employed. Specifically, zigzag nanotubes of the form [n,0] were selected, and the value of *n* was varied to tune the pore diameter. Nanotubes with *n* divisible by three were excluded, as these are metallic. The structures were generated using TubeGen v3.4,^34^ and a 1 × 1 × 4 supercell was subsequently optimized in CP2K at the PBE-D3­(BJ)/DZVP-MOLOPT-SR-GTH level of theory. For the MD simulations, the nanotube geometry was kept fixed. Thermal fluctuations of the nanotube radius are small relative to its diameter and are therefore not expected to significantly affect the results.

## 4 Results and Discussion

### 4.1 S_N_2 Reaction

#### 4.1.1 Static Reaction Profile

Figure 2 shows the energies of the S_N_2 reaction, in which CH_3_F reacts with F^–^. In the transition state of an S_N_2 substitution, the attacking and leaving groups, in this case two fluoride ions, are both partially bonded to carbon and located at comparable distances, such that the central carbon adopts a near-planar configuration before relaxing to a tetrahedral geometry in the product. The reaction profile was computed from static electronic-structure calculations at the xTB level of theory. The electronic energies and the corresponding free energies follow a similar profile, and the computed activation energy is 17 kJ mol^–1^.

**Figure 2:**
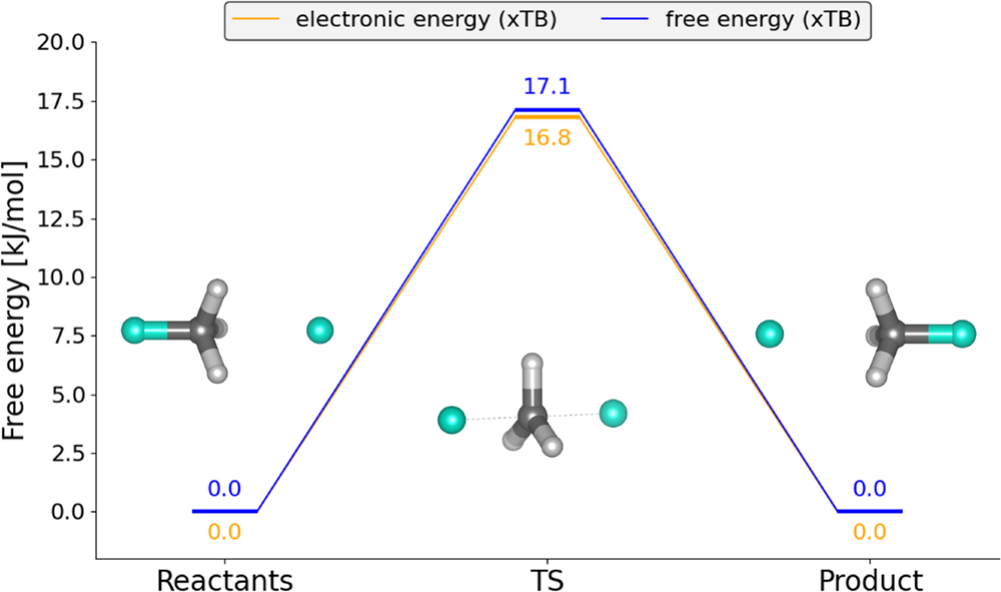
Static reaction profile for the S_N_2 model reaction CH_3_F + F^–^ → F^–^ + CH_3_F.

#### 4.1.2 Comparison of Metadynamics Methods for the S_N_2 Reaction

The same S_N_2 exchange reaction, CH_3_F + F^–^ → F^–^ + CH_3_F, was then modeled using CP2K with GFN1-xTB to enable molecular dynamics with longer simulation times. To limit the exploration of the free energy surface, restraining walls were applied at 4 Å on both C–F distances. Figure 3 compares the FES obtained with four metadynamics variants, plotted versus the CV defined as the difference between the breaking and forming C–F bond distances. The development of the CVs over time is shown in Figure S1 in the Supporting Information. Well-tempered metadynamics required 1000 ps to converge to a smooth surface. In contrast, OPES provided precise results already after 100 ps. OPES-Explore showed intermediate convergence speed but yielded an asymmetric free energy surface, which is incorrect given the symmetric nature of the reaction. The combination of OPES and OPES-Explore performed comparably to OPES alone, producing statistically reliable results without the asymmetry seen for OPES-Explore alone or the extended simulation times required by WT-MetaD. These observations highlight that the apparent accuracy of a metadynamics result depends strongly on methodological choices and sampling statistics. Even for a simple symmetric reaction, incomplete convergence can affect the free-energy landscape.

**Figure 3:**
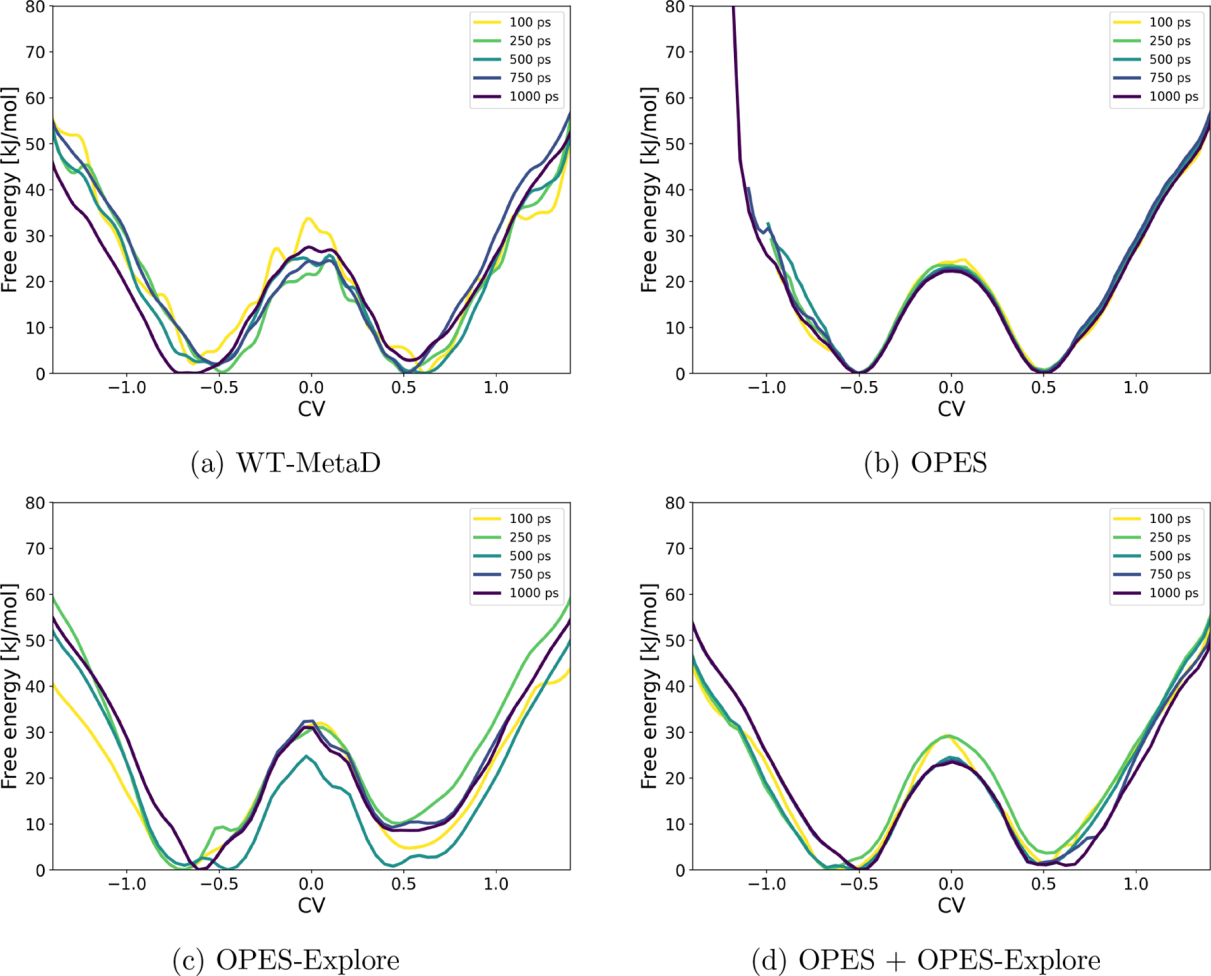
Evolution of the free-energy surface over time for the symmetric S_N_2 reaction CH_3_F + F^–^ → F^–^ + CH_3_F, using (a) well-tempered metadynamics, (b) OPES, (c) OPES-Explore, and (d) a combination of OPES and OPES-Explore. A wall at 4 Å on the C–F distances was applied in all simulations.

#### 4.1.3 Influence of Restraining Walls

Given that OPES + OPES-Explore performed best for this system, we used this metadynamics flavor to investigate the effect of wall placement on the reconstructed FES. All simulations were run for 500 ps. As shown in Figure 4, increasing the wall distance leads to broadened reactant and product basins, while the barrier height remains unchanged. At larger wall distances, the sampling quality deteriorates, as evidenced by the appearance of slightly asymmetric basins at 4.5 and 5.0 Å. Overall, the data suggest none or only a marginal increase in barrier height with increasing wall distance, while the basin width naturally increases as the system can explore a larger configurational space. Thus, in this case, the wall placement mainly determines the extent of sampling but does not significantly affect the reaction energetics.

**Figure 4:**
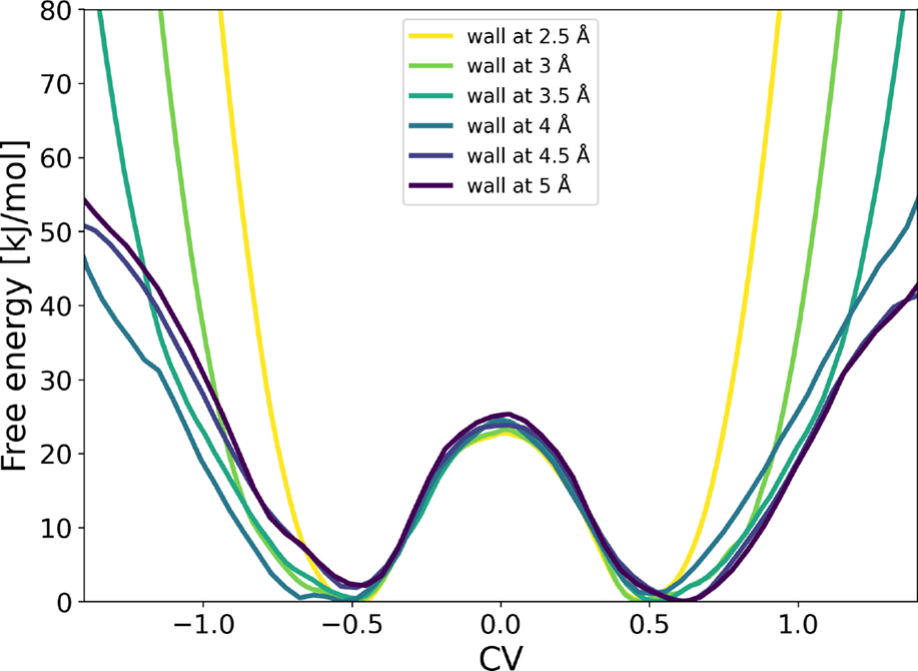
Free-energy surfaces from OPES + OPES-Explore simulations of the S_N_2 reaction with different positions of the restraining wall.

#### 4.1.4 Influence of Carbon Nanotube Diameter

A chemical way to constrain this reaction is to confine it within a carbon nanotube. Carbon nanotubes provide a suitable confinement environment because they do not form strong, directional interactions with CH_3_F. Figure 5 shows the free energy surface inside the carbon nanotube (7,0) with diameter 5.7 Å, CNT (8,0) with diameter 6.4 Å, and CNT (11,0) with diameter 8.7 Å. Calculations with CNT (10,0) were excluded due to consistent convergence issues. Each simulation was repeated three times using different starting geometries. Since OPES combined with OPES-Explore yielded the most stable and symmetric results for the reaction in isolation among all the tested methods, this combination was used for all CNT simulations.

**Figure 5:**
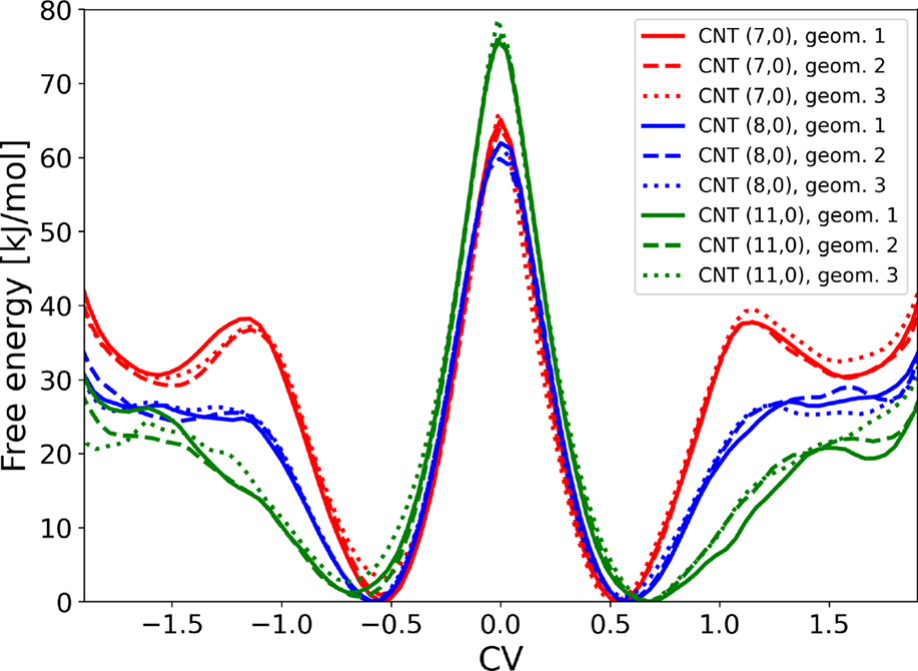
Free-energy surfaces of the S_N_2 reaction inside CNTs (7,0), (8,0), and (11,0) started from three different geometries each.

In all nanotubes, the absolute reaction barriers are higher than in the unconfined case, reflecting the stronger stabilization of the reactant and product states by the surrounding environment. This interpretation is supported by the interaction energies: for CNT (8,0), the transition state interacts with the nanotube by 608.5 kJ mol^–1^, whereas the sum of the separate interactions of F^–^ (544.1 kJ mol^–1^) and CH_3_F (160.6 kJ mol^–1^) with the CNT amounts to 704.7 kJ mol^–1^. Thus, the nanotube stabilizes the reactant (and product) states more strongly than the transition state, leading to an overall increase in the reaction barrier observed under confinement.

Clear differences emerge for the different CNT dimaters: The potential wells are narrowest for CNT (7,0), broader for CNT (8,0), and widest for CNT (11,0), reflecting the increasing spatial freedom available to the fluoride ion and CH_3_F molecule as the tube diameter grows.

The transition state region is similar for CNTs (7,0) and (8,0) but approximately 15 kJ mol^–1^ higher for CNT (11,0) across all initial geometries. Given the 6.4 Å inner diameter of CNT (8,0), the 3.6 Å F—F separation at the transition state would place one fluorine atom only about 1.4 Å from the wall if the F–C–F axis was oriented perpendicular to the tube axis, rendering such orientations sterically inaccessible (even more so in CNT (7,0)). In CNT (11,0), by contrast, the larger pore allows transition-state configurations in which the F–C–F axis lies perpendicular to the tube axis. This orientational freedom is absent in the narrower CNTs, where the reactants remain axially constrained and thus explore only a reduced subset of configurations. The enforced prealignment limits the extent of molecular reorientation required along the reaction path, decreases the configurational entropy of the prealigned reactant state, which increases its free energy and ultimately lowers the effective barrier height. This behavior represents a confinement-induced catalytic effect that depends on nanotube diameter.

The minimum around ± 1.5 Å corresponds to an ion–dipole configuration in which one fluorine atom is covalently bonded to the carbon atom, while the second fluoride remains at a distance of approximately 4.5 Å.

### 4.2 Diels–Alder Reaction

We chose the Diels–Alder reaction as prototypical reaction for studying confinement effects on pericyclic processes.

#### 4.2.1 Static Reaction Profile

Figure 6 shows that, at the GFN1-xTB level of theory, the equilibrium lies on the side of the Diels–Alder adduct. The product is energetically favored by approximately −131.2 kJ mol^–1^ in electronic energy and −57.1 kJ mol^–1^ in Gibbs free energy at 298 K relative to the separated reactants. The considerably larger difference between electronic and free energies compared to the S_N_2 reaction arises from the fact that two reactant molecules combine into one product, leading to more pronounced entropic changes. The following section compares how different metadynamics approaches describe this reaction in dynamical simulations.

**Figure 6:**
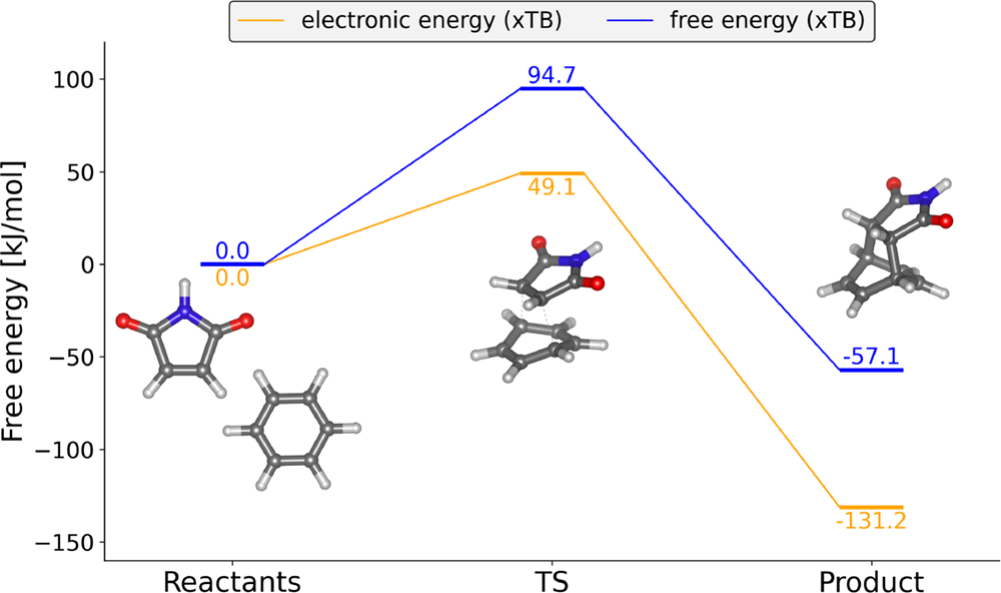
Static reaction profile of the Diels–Alder reaction between benzene and maleimide obtained at the GFN1-xTB level of theory.

#### 4.2.2 Comparison Metadynamics Methods for the Diels–Alder Reaction

The collective variable used here has been shown to yield free-energy surfaces consistent with those obtained from more elaborate CV definitions for this reaction.^35^ All simulations were initiated from the deepest free-energy basin corresponding to the product state, as recommended in a recent OPES overview publication.^36^ In the resulting surfaces, the product appears around a CV value of 1.5, while the reactant state is located at higher CV values (cf. Figure 7).

**Figure 7:**
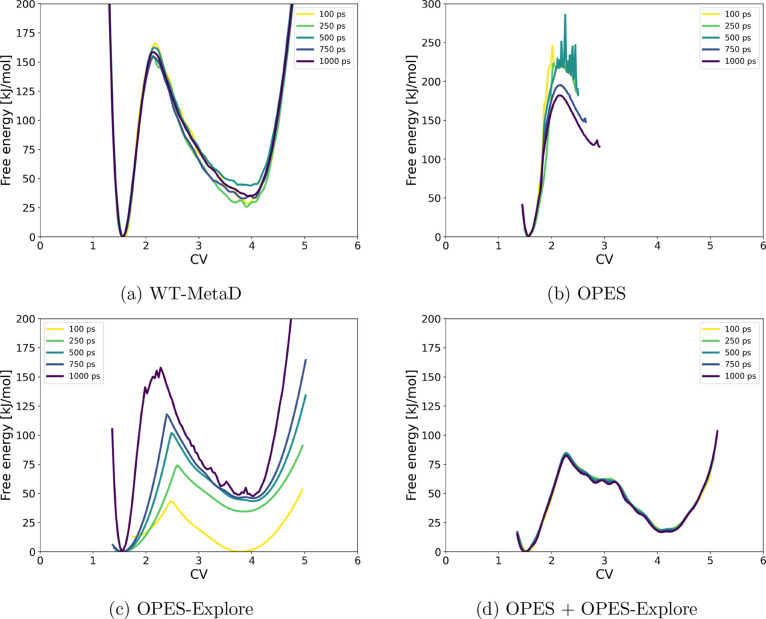
Evolution of the free-energy surface over time for the Diels–Alder reaction, using (a) well-tempered metadynamics, (b) OPES (note the different *y*-axis limit), (c) OPES-Explore, and (d) a combination of OPES and OPES-Explore. A wall constraint at 4 Å on the C–C distances involved in bond formation was applied in all simulations.

Among all methods tested, WT-MetaD showed the most reliable performance. It produced a well-defined transition region, converged smoothly, and yielded the activation barrier in best agreement with the static Gibbs free-energy one. The comparison between these results is qualitative, as metadynamics reconstructs the Helmholtz free energy in the NVT ensemble, whereas the static calculations with vibrational frequencies provide Gibbs free energies at 298 K. In contrast to WT-MetaD, the OPES simulation becomes trapped in metastable basins (cf. Figure S2 in the Supporting Information), a known limitation of this method arising from its high sensitivity to the choice of collective variables.^36^ In this context, deep-learning-assisted refinements of WT-MetaD have been shown to improve the resolution of complex Diels–Alder free energy landscapes, including cases with post-transition-state bifurcations, by enabling efficient evaluation of large numbers of configurations at a higher level of accuracy.^37^ However, such approaches were not considered in the present work. OPES-Explore alone leads to irregular free-energy profiles, an unsatisfactory result. The combined OPES + OPES-Explore scheme inherits the shortcomings of both approaches: while it reproduces the approximate shapes of the product basin near 1.5 Å and the reactant basin near 4 Å, the region between them is incorrect, and the method failed to deliver consistent profiles when different wall constraints are applied (see Figure S4 in the Supporting Information). Thus, owing to its reliable convergence and agreement with the static profile, WT-MetaD was employed for all subsequent simulations of the Diels–Alder reaction.

#### 4.2.3 Walls

As for the S_N_2 reaction, the effect of applying an artificial wall potential in the metadynamics simulation was also examined for the Diels–Alder system (cf. Figure 8 for the FES and Figure S3 for the time-development of the CVs). In these simulations, restraining walls were placed at increasing C–C distances to limit the exploration of configurational space. The wall position has no large impact on the transition-state barrier: in all cases, the barrier from the product basin to the transition state remains close to 150 kJ mol^–1^, in excellent agreement with the static value of 151.8 kJ mol^–1^.

**Figure 8:**
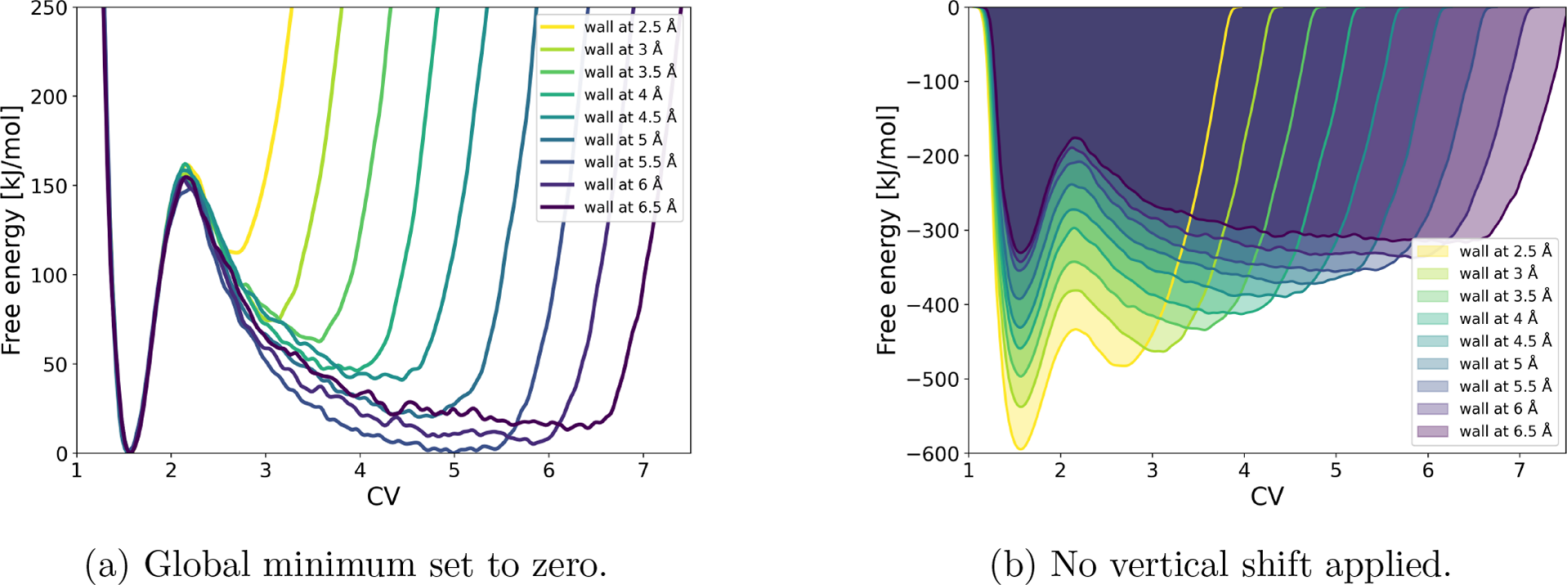
Free-energy surfaces from well-tempered metadynamics simulations of the Diels–Alder reaction with different restraining-wall positions.

When the wall position is moved outward, the reactant basin becomes progressively broader and deeper. This trend arises because the two separated reactants can explore progressively more configurations, which increases their configurational entropy and thereby lowers their Helmholtz free energy, a well-established volume dependence.^38^ In contrast, the product, being a single molecule, is only weakly affected by the position of the wall. As a result, the intrinsic electronic stabilization of the product is gradually offset by the growing entropic contribution of the reactants, and both states become approximately isoenergetic at a wall distance of 5.5 Å. At even larger wall distances, the reactant basin becomes shallower again. The deviation from the static calculation, where the product is clearly favored, originates from the fact that the combined entropic contribution of the two reactants is not captured in the static treatment.

This illustrates how confinement-induced entropic effects can reshape free-energy profiles and, accordingly, how restraining walls in metadynamics can modify the resulting free energies.

#### 4.2.4 Inside Carbon Nanotubes

Figure 9 shows the free energy surfaces of the Diels–Alder reaction inside carbon nanotubes of types (11,0), (13,0), (14,0), and (16,0). All free energy surfaces are shifted such that the minimum of the product basin corresponds to 0 kJ mol^–1^. This choice is arbitrary as only the relative differences are meaningful; Figure S5 in the Supporting Information presents the same profiles where the global minimum is set to 0. Table S1 in the Supporting Information lists the integrated free-energy values.

**Figure 9:**
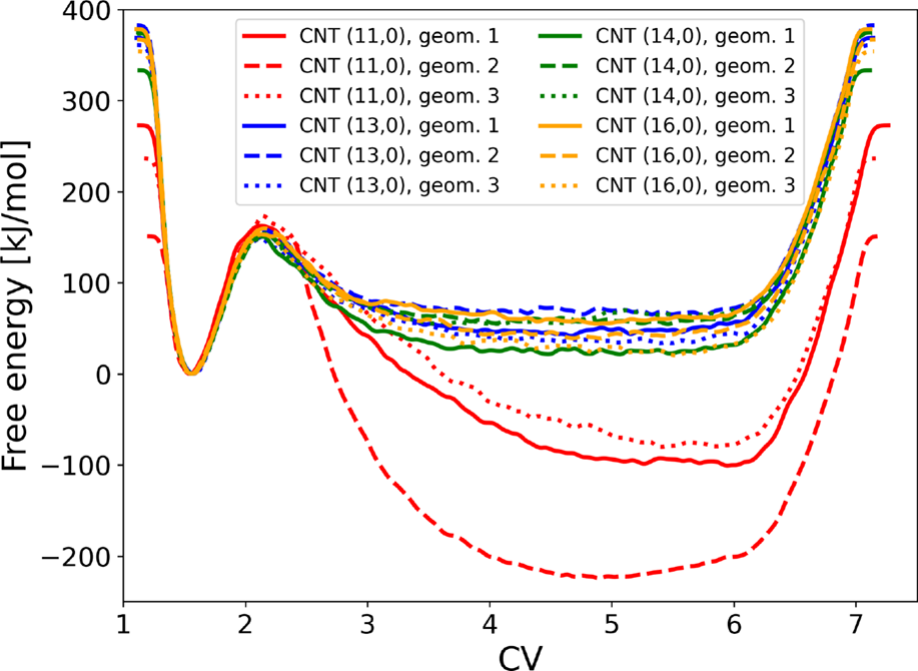
Free-energy surfaces of the Diels–Alder reaction inside carbon nanotubes of different diameters.

In Figure 9, one can see that the barrier from the product basin (around CV 1.5) to the transition state remains essentially unchanged across all tubes. The effect on the relative stability of the reactant basin is more pronounced, analogously to the behavior observed with the artificial wall constraint. For CNT (11,0), the barrier from the reactant basin side is significantly higher, reflecting the difficulty of forming the transition state within the limited space of the narrow pore. This behavior is also evident in the CV versus time trajectories shown in Figure S6, where starting geometry 2 inside CNT (11,0) never returns to the product state within the simulated time window. The results also indicate a non-negligible degree of statistical uncertainty on the reactant side, as the free-energy surface depends on the initial configuration. Throughout all simulations, the reacting molecules remain centered within the tube rather than adsorbing on the wall (cf. Figure 10), confirming that strong interactions with the tube wall do not play a major role.

**Figure 10:**
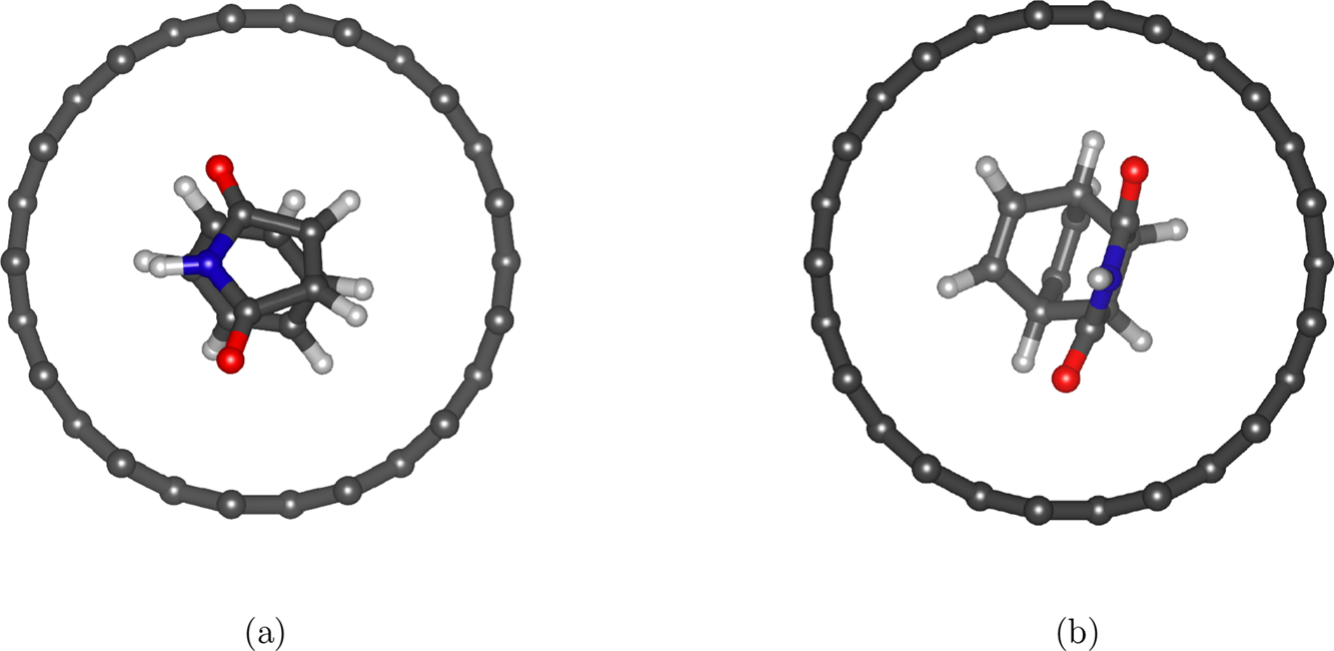
(a) Reactants optimized in CNT (13,0) and (b) product optimized in CNT (13,0).

## 5 Conclusions

In this study, we examined how physical and methodological confinement influence reaction free-energy surfaces obtained from different metadynamics methods, using two prototypical reactions, an S_N_2 substitution and a Diels–Alder cycloaddition, as test cases. The purpose was 2-fold: (i) to assess how different forms of confinement affect the resulting free energy profiles and (ii) to compare the performance of several metadynamics approaches for confined reactions.

Regarding the influence of confinement, the S_N_2 reaction shows a clear response to geometric restriction in a carbon nanotube. Confinement within the tube lowers the activation barrier as the diameter decreases, because the restricted geometry enforces axial prealignment of the reactants. In contrast, artificial confinement through restraining walls on the C–F distance in metadynamics leaves the barrier largely unchanged but obviously reduces the widths of the reactant and product basins. For the Diels–Alder reaction, confinement effects become pronounced only in the narrow CNT­(11,0), which can easily accommodate the two reactant molecules but is too small for the product. For the larger CNTs the barrier remains essentially constant. The dominant energetic contribution to the activation energy arises from the dearomatization and structural distortion of benzene required for cycloaddition, which is not substantially alleviated by the tube environment. As a result, the barrier inside the nanotube and without nanotube remains comparable.

The comparison of metadynamics approaches shows that no single method performs best across both reactions. For the symmetric S_N_2 reaction, the combined OPES + OPES-Explore scheme produced the most accurate and symmetric free-energy surface. For the Diels–Alder reaction, well-tempered metadynamics yielded the most stable and reproducible profiles. This difference highlights that the optimal method depends on the system. In both cases, OPES-Explore alone did not provide statistically reliable results, highlighting that it is valuable mainly for exploration and locating metastable states rather than for generating converged free energy surfaces.

Finally, although confinement might be expected to simplify sampling by reducing the accessible configurational space, in practice it introduces additional complexity. This must be considered carefully when selecting collective variables, walls, and when interpreting free energy surfaces obtained under confined conditions.

## Supplementary Material



## Notes

CV evolution over time for the S_N_2 reaction reaction, CV evolution over time for the Diels–Alder reaction, OPES + OPES-Explore free-energy profile for the Diels–Alder reaction, WT-MetaD free-energy profile for Diels–Alder reaction in carbon nanotubes referenced to the global minimum, collective variable development of Diels–Alder WT-MetaD simulation in various CNTs, and integrated free energy in reactant and product basin for Diels–Alder reaction in carbon nanotubes (PDF)
