# Supplementary material for: Free-Energy Profiles of Confined Reactions: Influence of Confinement Type and Challenges for Metadynamics Methods
Source: ACS Phys Chem Au. 2026 Apr 24;6(3):493–502. doi: 10.1021/acsphyschemau.5c00151 (PMC13220192; doi:10.1021/acsphyschemau.5c00151)
Supplement: Supplementary file 1 [file pg5c00151_si_001.pdf]

Supporting Information: Free-energy profiles of confined reactions: influence of confinement type and challenges for metadynamics methods

Michelle Ernst*,† and Jürg Hutter‡

†*Institute of Geological Sciences, University of Bern, Baltzerstrasse 1+3, 3012 Bern, Switzerland.*

‡*Department of Chemistry, University of Zurich, 8057 Zürich, Switzerland.*

E-mail: michelle.ernst@unibe.ch

1 CV evolution over time for the S_N2 reaction

Figure S1 shows the evolution of the collective variable over time for all the investigated metadynamics approaches as discussed in *section 4.1.2 Comparison of metadynamics methods* in the main article.

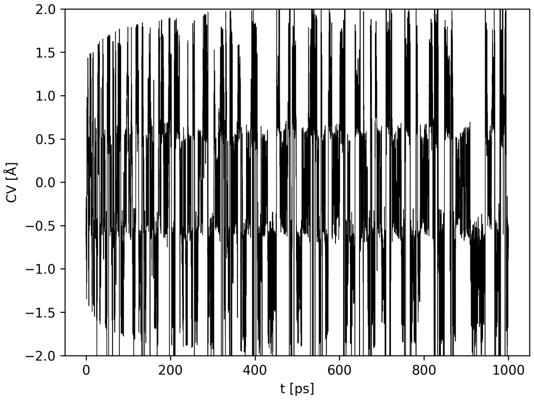

(a) WTMetaD

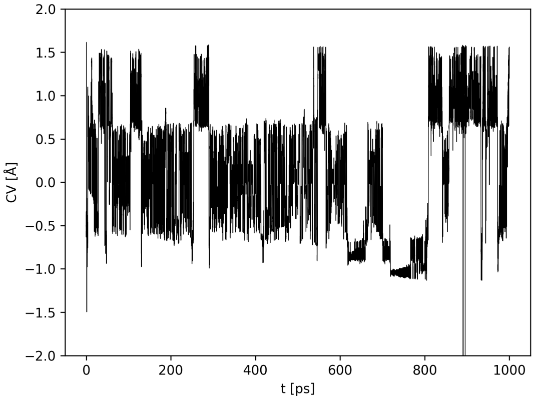

(b) OPES

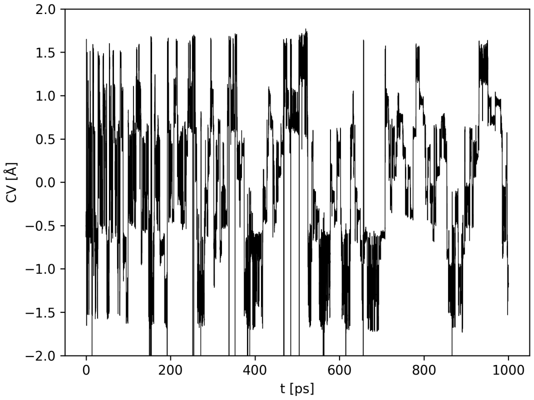

(c) OPES-Explore

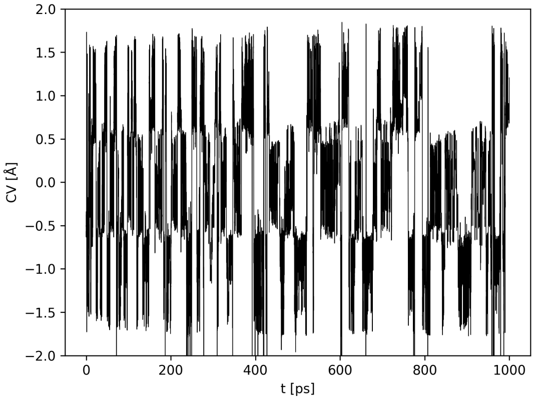

(d) OPES + OPES-Explore

Figure S1: Evolution of the collective variable $0.5\,d_1 - 0.5\,d_2$ over time for the $\mathrm{S_N2}$ reaction, using (a) well-tempered metadynamics, (b) OPES, (c) OPES-Explore, and (d) OPES + OPES-Explore. A restraining wall at 4 Å was applied to the C–C distances involved in bond formation in all simulations.

2 CV evolution over time for the Diels–Alder reaction

Figure S2 shows the evolution of the collective variable over time for all metadynamics approaches as discussed in *section 4.2.2 Comparison metadynamics methods* in the main article.

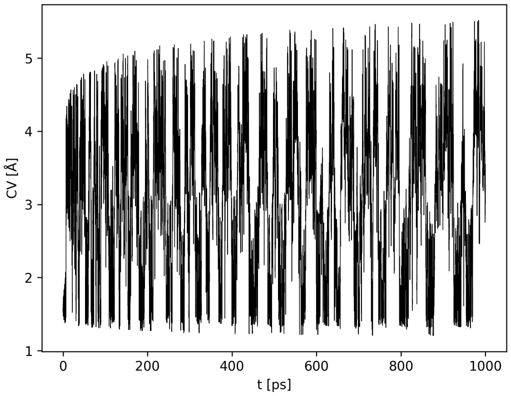

(a) WTMetaD

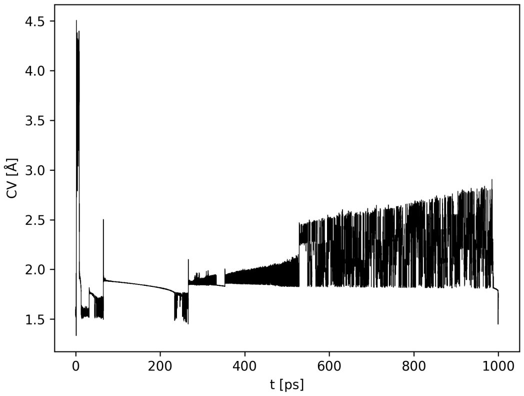

(b) OPES

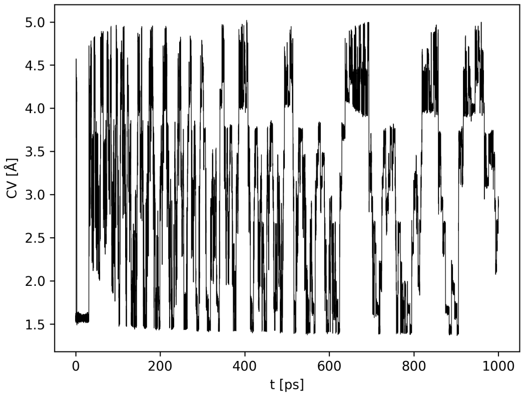

(c) OPES-Explore

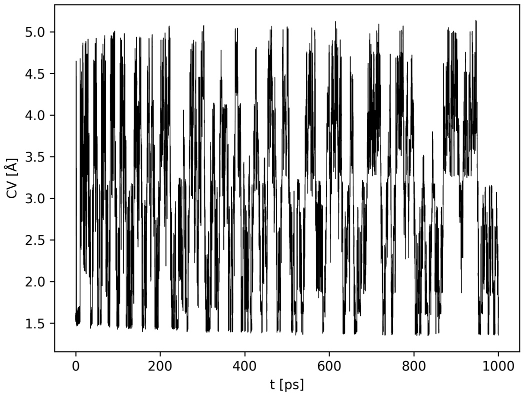

(d) OPES + OPES-Explore

Figure S2: Evolution of the collective variable $0.5\,d_1 + 0.5\,d_2$ over time for the Diels–Alder reaction, using (a) well-tempered metadynamics, (b) OPES, (c) OPES-Explore, and (d) OPES + OPES-Explore. A restraining wall at 4 Å was applied to the C–C distances involved in bond formation in all simulations.

Figure S3 shows the evolution of the collective variable over time for the well-tempered metadynamics simulations with different wall positions as discussed in *section 4.2.3 Walls* in the main article.

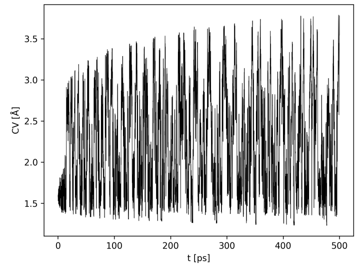

(a) wall 2.5 Å

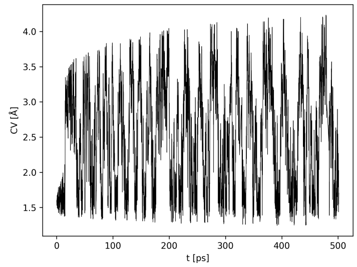

(b) wall 3.0 Å

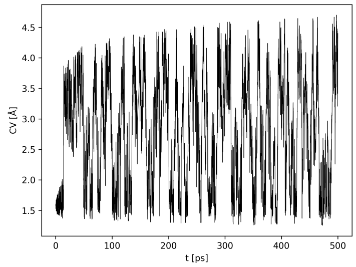

(c) wall 3.5 Å

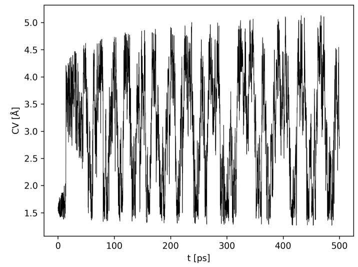

(d) wall 4.0 Å

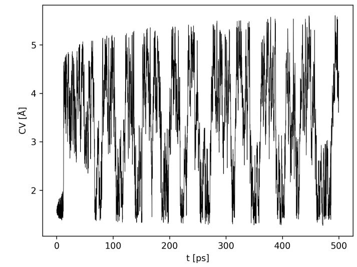

(e) wall 4.5 Å

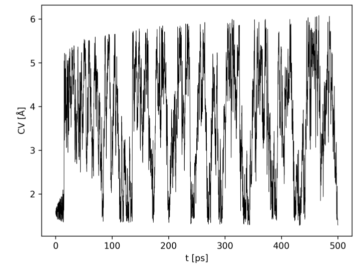

(f) wall 5.0 Å

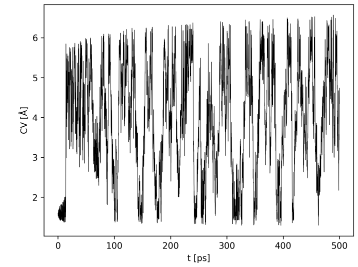

(g) wall 5.5 Å

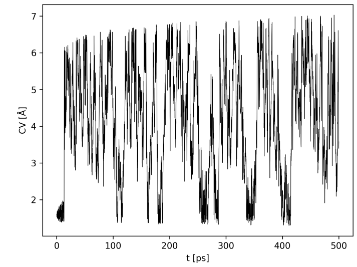

(h) wall 6.0 Å

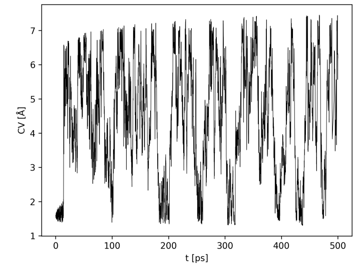

(i) wall 6.5 Å

Figure S3: Evolution of the collective variable as a function of time for the Diels–Alder reaction using well-tempered metadynamics with different restraining wall positions: (a) 2.5 Å, (b) 3.0 Å, (c) 3.5 Å, (d) 4.0 Å, (e) 4.5 Å, (f) 5.0 Å, (g) 5.5 Å, (h) 6.0 Å, and (i) 6.5 Å.

3 OPES + OPES-Explore for the Diels–Alder reaction

Figure S4 shows the free-energy profiles obtained with OPES + OPES-Explore for different wall positions. The resulting surfaces display substantial variability, indicating that the combined scheme does not produce robust or reproducible free-energy estimates for this reaction.

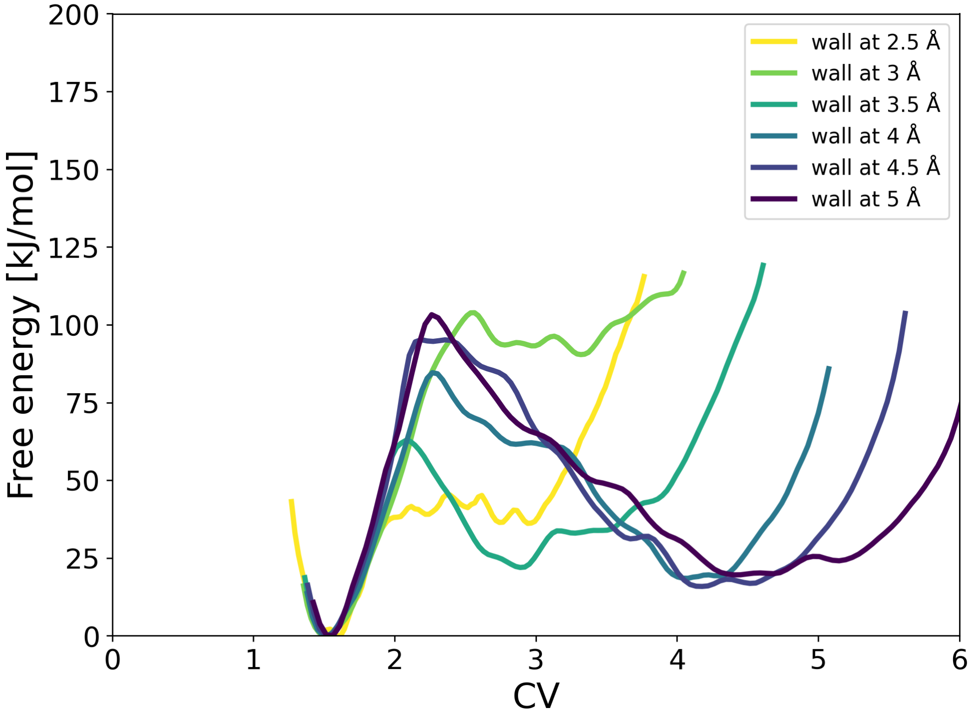

Figure S4: Free energy surfaces from OPES + OPES-Explore simulations of the Diels–Alder reaction with different wall positions.

4 Free-Energy surfaces referenced to the global minimum

Figure S5 shows the free-energy surfaces of the Diels–Alder reaction inside carbon nanotubes of types (11,0), (13,0), (14,0), and (16,0). All free energy surfaces are shifted such that the global minimum corresponds to 0 kJ mol^{-1}.

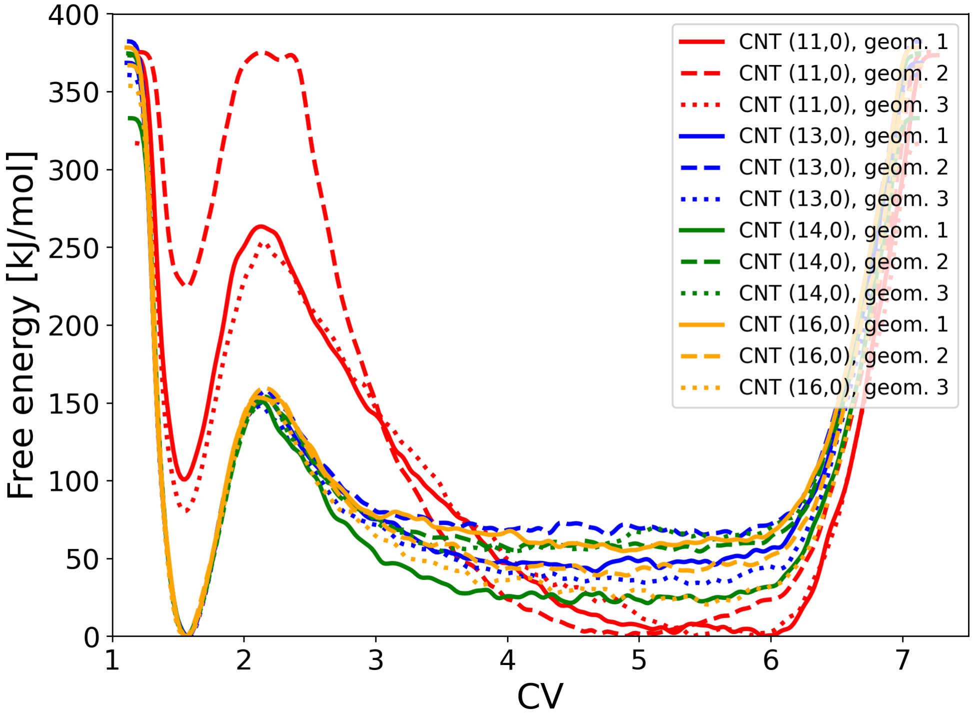

Figure S5: Free energy surfaces of the Diels–Alder reaction inside carbon nanotubes of different diameters.

5 Collective variable development in carbon nanotubes

Figure S6 shows the development of the collective variable in carbon nanotubes during the well-tempered metadynamics simulation.

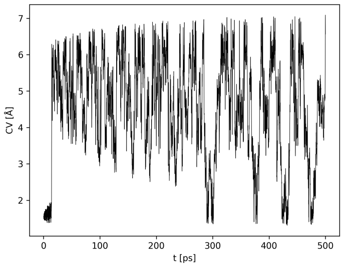

(a) CNT (11,0), geom1

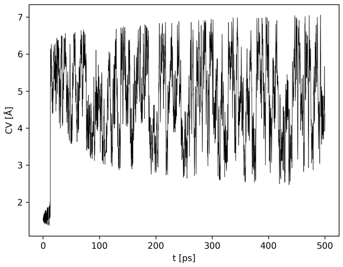

(b) CNT (11,0), geom2

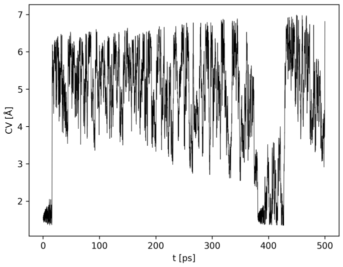

(c) CNT (11,0), geom3

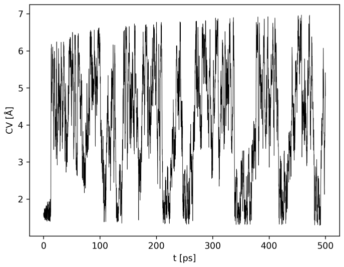

(d) CNT (13,0), geom1

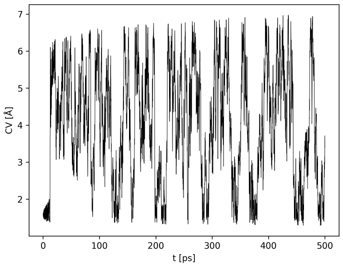

(e) CNT (13,0), geom2

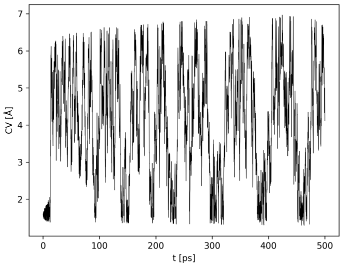

(f) CNT (13,0), geom3

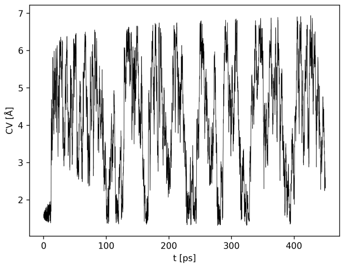

(g) CNT (14,0), geom1

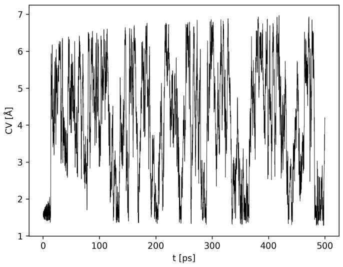

(h) CNT (14,0), geom2

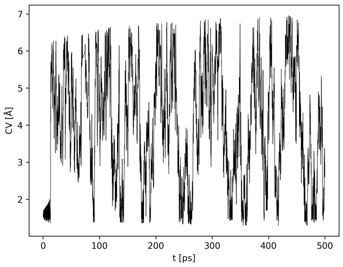

(i) CNT (14,0), geom3

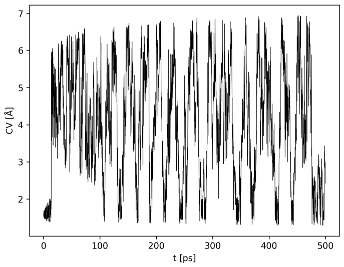

(j) CNT (16,0), geom1

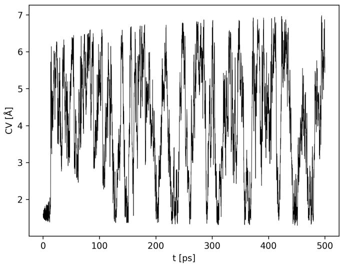

(k) CNT (16,0), geom2

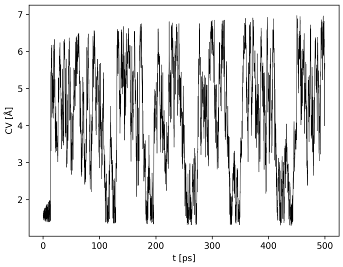

(l) CNT (16,0), geom3

Figure S6: Evolution of the collective variable $0.5\,d_1 + 0.5\,d_2$ over time for the Diels–Alder reaction in CNTs of different diameters simulated with WT-MetaD.

6 Integrated free energy in reactant and product basin for Diels–Alder reaction in carbon nanotubes

To quantify the thermodynamic stability of the reactant and product states, the free energy surfaces were integrated within the respective basins for each carbon nanotube system. Table S1 summarizes the resulting values for all examined geometries and nanotube types.

Table S1: Integrated free energy values for the Diels–Alder reaction inside carbon nanotubes of different diameters simulated with WT-MetaD. The integration was carried out separately over the product and reactant basins of the free energy surface up to the free energy of the transition state.

System	Product basin [$kJ\,mol^{-1}$ Å]	Reactant basin [$kJ\,mol^{-1}$ Å]
CNT (11,0), geom 1	66.85	726.51
CNT (11,0), geom 2	63.08	1153.97
CNT (11,0), geom 3	76.83	671.63
CNT (13,0), geom 1	64.33	339.35
CNT (13,0), geom 2	67.36	296.80
CNT (13,0), geom 3	62.43	353.95
CNT (14,0), geom 1	62.81	409.57
CNT (14,0), geom 2	66.37	317.22
CNT (14,0), geom 3	65.96	309.09
CNT (16,0), geom 1	63.48	301.60
CNT (16,0), geom 2	67.47	371.19
CNT (16,0), geom 3	66.02	398.97
